# A clinical update on massive ovarian oedema – a pseudotumour?

**DOI:** 10.3332/ecancer.2013.318

**Published:** 2013-05-14

**Authors:** RS Praveen, VR Pallavi, K Rajashekar, A Usha, K Umadevi, UD Bafna

**Affiliations:** 1 Department of Gynaecologic Oncology, Kidwai Memorial Institute of Oncology, Bangalore, India; 2 Department of Pathology, Kidwai Memorial Institute of Oncology, Bangalore, India

**Keywords:** Massive ovarian Oedema, pseudotumour of ovary, fertility-sparing surgery, ovarian drilling, ovarian reconstruction

## Abstract

**Objective:**

Benign massive ovarian oedema is a rare clinical entity arising from the ovaries, and it poses a significant clinical challenge as it can be easily mistaken for neoplasm. Due to the lack of pathognomonic clinical features or characteristic hallmarks on non-invasive diagnostic modalities and the dependence on the final histopathology, the efforts of the surgeon have been deviated from performing fertility-sparing surgery on young women. The lack of standardised guidelines due to the rarity of this condition calls for a review of the literature to enable the clinician to formulate treatment guidelines.

**Methods and Material:**

A Medline search on the PubMed database for literature published in English from 1969 to 2011 was done using the keywords ‘massive ovarian oedema, massive ovarian oedema case report or case series, and pseudotumour of ovary’. A total of 177 women who had undergone a variety of treatments were retrieved. We also report the management options we used for four women presenting to us between August 2000 and October 2011, as well as a review of the literature.

**Result:**

A total of 177 cases of massive ovarian oedema were identified. Out of these cases 151 (85.3%) were primary massive ovarian oedema; secondary massive ovarian oedema was identified in 26 (14.7%) cases. A salpingo-oophorectomy was done in 145 (81.9%) cases, 12 (6.8%) cases had an abdominal hysterectomy with bilateral salpingo-oophorectomy. A total of 76 (42.9%) cases intraoperatively were found to have ovarian torsions, and one patient with primary massive ovarian oedema had ascites. Conservative treatment was carried out in 20 (11.3%) patients; 14 of these had a wedge biopsy with frozen section and with or without ovarian suspension, one patient had diagnostic laparotomy, and five cases had only ultrasonographic or magnetic resonance imaging monitoring and symptomatic treatment. The four cases treated at the regional cancer institute from 2000 to 2011 revealed that the first three cases had salpingo-oophorectomy and the fourth case received a successful conservative treatment.

**Conclusion:**

The majority of massive ovarian oedemas will respond to judicious use of intraoperative wedge resection and frozen section for the confirmation of diagnosis. The detorsion and transfixation of the ovary or partial debulking and drainage of fluid accumulated in the cyst may be more appropriate to preserve hormonal function and fertility in these young women.

## Introduction

Massive ovarian oedema is a rare solid tumour-like condition seen in young women. The term ‘massive ovarian oedema’ was first described by Kalstone in 1969 [1] and the World Health Organization defined it as an accumulation of oedema fluid within the ovarian stroma separating normal follicular structures [2]. The most commonly affected are young women in their reproductive stage, but a singular cases of a six-month-old infant and a postmenopausal woman have also been reported [3, 4]. Massive ovarian oedemas can involve one or both the ovaries [5, 6], and it has also been observed during pregnancy [7]. The most common presenting symptoms are pain, distension or mass in the abdomen, infertility, and in some cases irregular vaginal bleeding [2–7]. The masculinisation features are frequently seen in many adult cases, and rarely a small number of cases are known to present with precocious puberty [3, 8–10].There are at least two types of massive ovarian oedema: primary, without concomitant pathology, and secondary, superimposed on already altered ovaries [11]. The most favoured hypothesis for the aetiology is the development of massive oedema as a result of the disturbed venous and lymphatic circulation following complete or partial torsion of the mesovary, but not the arterial blood flow [2–6, 8–10]. As a result, there is stromal cell luteinisation in the oedematous ovary, occurring as a response to torsion and subsequent ischaemia. The stromal cells have positive oestrogen progesterone receptors and may undergo stimulatory changes responsible for the hormonally related symptoms often found associated with massive ovarian oedema [3, 4, 8–10]. Derangement of a local paracrine factor, such as insulin-like growth factor, epidermal growth factor, or cytokines, was an alternate explanation proposed by Eden *et al *[9]. Massive ovarian oedema is commonly associated with normal levels of lactate dehydrogenase levels and tumour markers [3–11]. Secondary massive ovarian oedema rarely occurs in diseased ovaries with associated benign cysts and tumours [5, 12–17], the drugs used for ovulation induction [18] and malignancies [19–21]. Few reported cases of raised Ca-125 levels with or without raised lactate dehydrogenase levels in massive ovarian oedema with Meig’s syndrome, fibrothecomas, and so on are available in the literature [16, 17].

## Radiological features

A definitive preoperative diagnosis of massive ovarian oedema is often difficult and the ultrasonographic findings are variable and not diagnostically accurate. The ultrasound findings in the majority of the cases have been reported as a heterogeneous complex ovarian mass, which is non-specific and can mimic neoplasia [4, 6]. However, Umesaki *et al *[22] described the ultrasonographic features that a solid ovarian tumour with multiple peripheral ovarian follicles with or without endometrial hypertrophy may indicate the possibility of massive ovarian oedema. Furthermore, Umesaki *et al *[23] described in another case report, a successful preoperative diagnosis of massive ovarian oedema aided by comparative imaging study using magnetic resonance imaging (MRI) and ultrasonography; as normal ovarian follicles are pressed towards the peripheral cortical area of the ovary by oedema fluid accumulated within the ovarian stroma. Similarly, Hall *et al* also report that by using MRI, they have demonstrated that multiple ovarian follicles situated around the periphery of the cortex of the enlarged ovary are the most characteristic of massive ovarian oedema [24]. This appearance corresponds to the World Health Organization’s definition of massive ovarian oedema, namely, as ‘an accumulation of oedema fluid within the ovarian stroma separating normal follicular structures’ [2]. The finding of multiple ovarian follicles located at the peripheral cortex of an enlarged ovary is thus a very important diagnostic indicator of massive ovarian oedema.

## Morphological features

**Figure 1:**
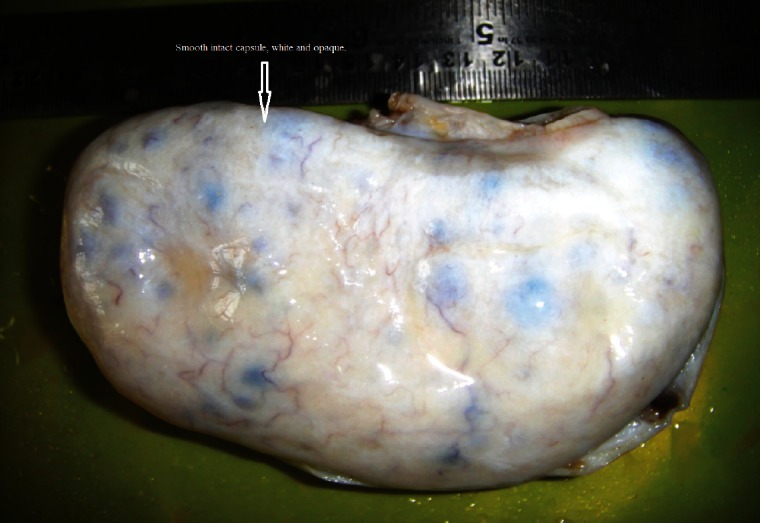
The external surface of the massive ovarian oedema is usually white and opaque.

**Figure 2:**
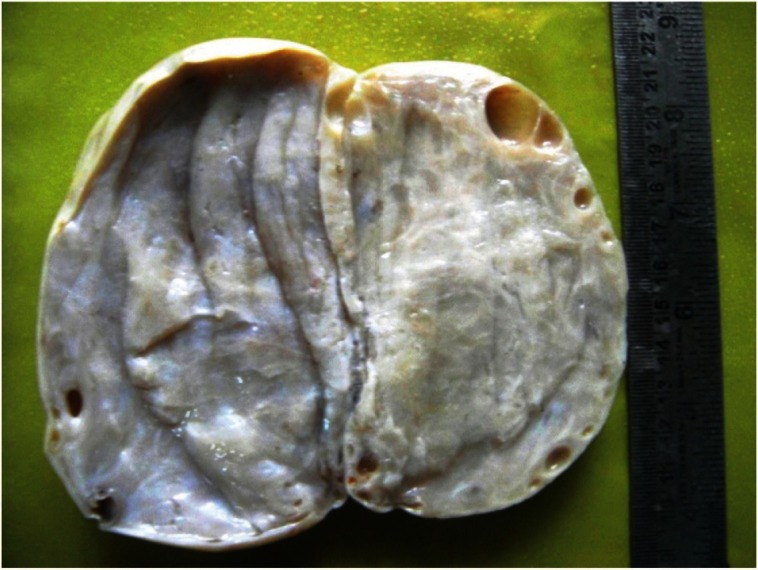
The sectioned surface typically exudes watery fluid after cutting with a knife due to the pressure of the edema.

The morphological recognition of the lesion is fairly simple. The external surface is usually white and opaque. The sectioned surface typically exudes watery fluid after cutting with a knife due to the pressure of the oedema [2] (Figures 1 and 2).

## Histological features

**Figure 3:**
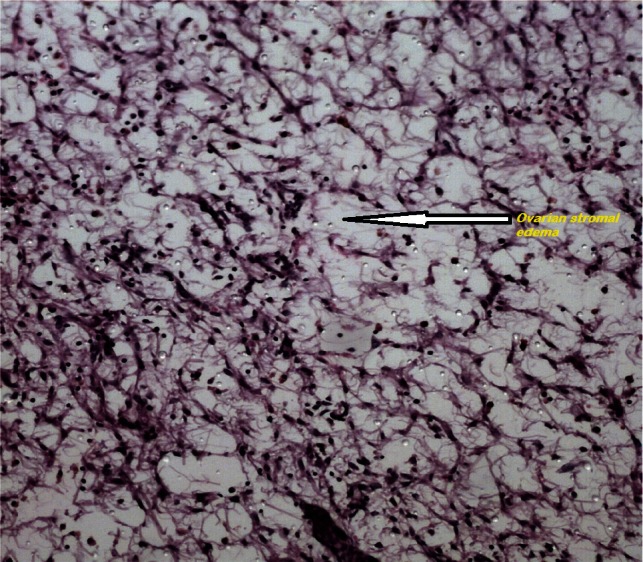
An ovarian stroma with marked oedema, at. H&Ex400.

**Figure 4:**
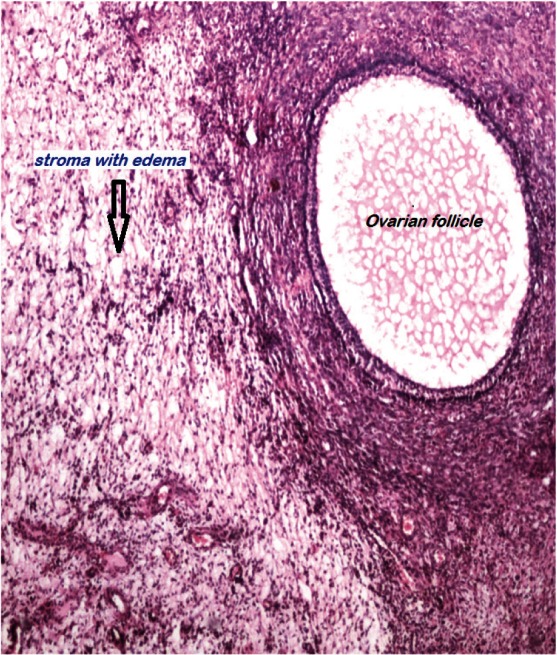
Ovarian stroma with edema. A follicle is also seen. H&EX100.

Histologically, an oedematous and hypocellular ovarian stroma is present, and the ovarian architecture is preserved. The outer cortex is thickened and fibrotic. A cluster of luteinised stromal cells is present in the oedematous stroma in the minority of cases, especially those that have endocrine symptoms [2] (Figures 3 and 4). Necrosis and haemorrhage are unusual [2, 25]. Focal stromal luteinisation has been noted in some of the studied cases and is thought to be a mechanical process induced by stretching of the stromal cells [3]. Immunohistochemical studies have shown phenotypic modulation of fibroblasts in massive ovarian oedema [26].

Primary massive ovarian oedema is a rare benign tumour-like condition of the ovary and can be easily mistaken for malignancy, in which case the appropriate treatment will be removal of the whole ovary. However, as this condition is a benign enlargement of the ovary, it warrants a conservative treatment. We emphasise the need for a high degree of clinical suspicion preoperatively in this rare disorder, and the knowledge gained from the literature helped us to focus on fertility-sparing surgery in these young women, whose ovaries need to be conserved for further reproductive and hormonal functions.

## Methods

A Medline search on Pub Med database since 1969 using the keywords ‘‘massive ovarian edema, massive ovarian edema case report or case series and a pseudotumor of ovary’’ found around 177 cases reported: 85 articles reporting a single case, 11 articles reporting two cases, four articles reporting three cases, and single articles each reporting four, five, six, eighteen and twenty five cases respectively were identified. The relevant articles in English with a specific interest in ovarian oedema were reviewed. The references of these articles generated also enabled a widening of the reference pool.

## Results

One hundred and fifty-one of 177 (85.3%) were primary massive ovarian oedema, of which 5 (2.8%) were associated with pregnancy. Secondary massive ovarian oedema was identified in 26 of the 177 (14.7%) women who were associated with a variety of clinical conditions such as pelvic fibromatosis, Meig’s syndrome, mucinous cyst adenoma of ovary, polycystic ovarian disease, clomiphene citrate administration, and contralateral ovarian mature teratoma, lymphoma, carcinoma stomach, uterine cervical cancer, and lung cancer in 8, 5, 3, 3, 2 and 1 cases in each category, respectively. The age group ranged from a neonate at six months of age to postmenopausal women up to 60-years-old. There were no pathognomonic clinical features, as all the children and women presented with abdominal pain and abdomino-pelvic mass detected either by clinical examination or by non-invasive radiological methods such as ultrasound, computed tomography (CT) scan or MRI. Variable clinical features such as ascites, hydrothorax, abnormal vaginal discharge, anorexia, fever and haemoptysis were more predominant in the secondary massive ovarian oedema except for one woman with ascites in the primary group. Virilisation was seen in 37 of 177 (20.9%) of women. Ovarian torsion was encountered in 76 of 177 (42.9%) intraoperatively.

## Regional cancer institute experience

**Figure 5 and 6:**
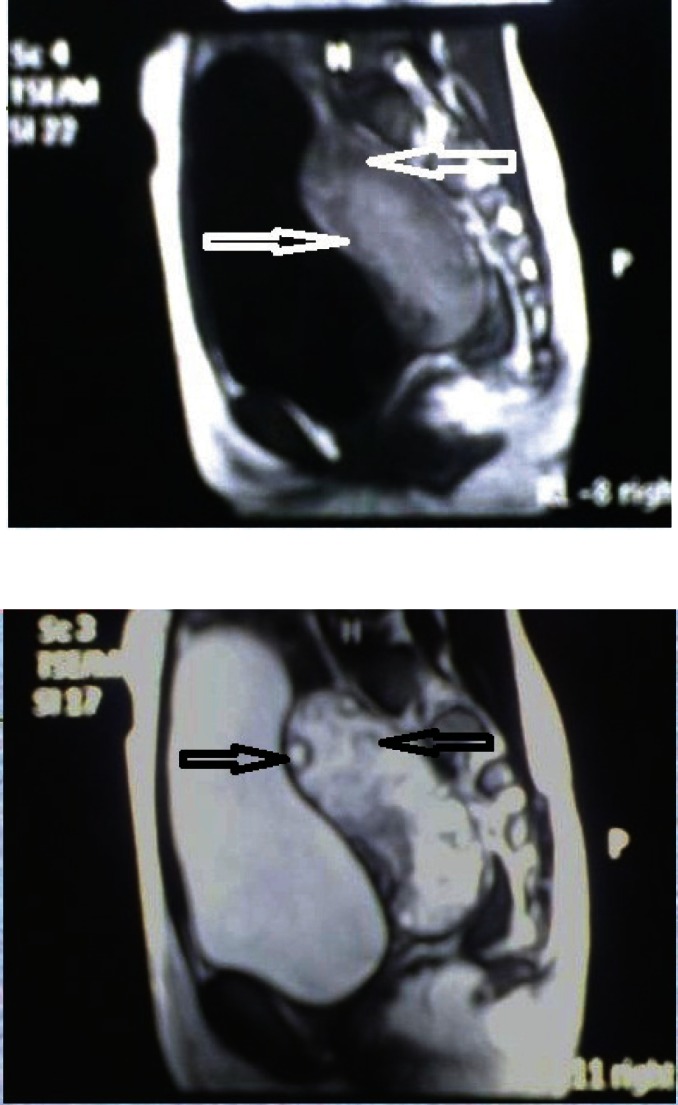
MRI of T1 & T2 weighted image isodense to hypointense capsule. The image did not show any fat component within and multiple normal ovarian follicles(arrows) noted in the periphery of the mass.

We present the study of four cases of massive ovarian oedema managed at our regional cancer institute, from August 2000 to October 2011. The youngest patient was an eight-year-old premenarcheal girl, the oldest was a 26-year-old multipara and two were menarcheal girls aged 11 and 17 years. All four of them presented with abdominal pain, bimanually palpable adnexal masses, and case 2 had signs of virilisation. Ultrasonography revealed the presence of unilateral adnexal multicystic lesion in case 1 (R) and 3 (L) with characteristic peripheral orientation of multiple cysts, bilateral lesions with solid and cystic areas in cases 2 and 4 as described in Table 2. Figure 5 (case 1) shows the MRI characterisation of the right adnexal lesion measuring 8.2 cm × 3.2 cm × 5.2 cm that was isodense to hyperintense on both T1- and T2-weighted sections and contained well-defined hypointense capsule and did not show any fat component within. All the four patients had normal values of serum beta-human chorionic gonadotrophin (S-βhcg), alpha feto-protein (AFP), cancer antigen-125 (CA-125) and lactate dehydrogenase (LDH) levels.

All the four cases had laparotomies as malignancy was suspected. A frozen section was performed in all four cases that revealed massive ovarian oedema and was confirmed as primary massive ovarian oedema in all the four specimens by final histopathological examination (Figures 3 and 4). A search for intra-abdominal metastatic deposits did not contribute significantly, see Table 3.

## Discussion

Massive ovarian oedema can occur as a primary or secondary oedema. Primary massive ovarian oedema is most commonly diagnosed, which is seen in more than 85% of reported cases. Primary oedema occurs in a normal ovary with torsion or twisting of the ovarian pedicle to the extent that it interferes with the venous blood supply without affecting the arterial flow leading to oedema. Incomplete or intermittent torsion can occur. Secondary massive ovarian oedema occurs in a diseased ovary, such as when there is an ovarian mass or cyst-like ovarian capillary haemagioma [12], mucinous and serous cyst adenomas [13, 14], mature cystic teratoma [15], Meig’s syndrome [16], ovarian fibrothecoma [17], and polycystic ovary [5]. And malignancies, causing to lymphatic permeation by metastatic carcinoma from the uterine cervix [19], gastric carcinoma [20], and lymphangitis carcinomatosa [21]. Secondary to the drugs used for ovulation induction [18]. Young *et al *explained that fibromatosis and massive oedema of the ovary are possibly related entities, as discussed in a report of 14 cases of fibromatosis and 11 cases of massive oedema [27]. The similar age range and clinical manifestations of these two processes and the overlap in their histological features suggest that they are closely related and may reflect differing morphologic expressions of the same underlying disorder. Some of the cases of massive oedema, however, may result from the development of stromal oedema in ovaries involved by hyperthecosis [5].

Primary massive ovarian oedema is a rare clinical condition, occurring in young women, with an absence of any pathognomonic clinical features, as it resembles an ovarian cyst with or without torsion, pain in the abdomen being the commonest presentation. Radiological imaging in most of the situations can be ambiguous, with addition of tumour markers such as S-βhcg, LDH, CA-125, AFP; the differential diagnosis can be scaled down, differentiating the condition from dysgerminomatous and mixed germ cell tumours. However, an intraoperative frozen section can assist in performing a fertility-sparing surgery, which will have a bearing on her future reproduction.

An extensive search of the literature regarding the management of massive ovarian oedema reveals that, the majority of the women had been over treated with salpingo-oophorectomy on the mistaken pretext of an association with malignancy [2–10], similar to our institutional experience of the first three patients who also underwent a unilateral salpingo-oophorectomy. Data supporting conservative treatment in massive ovarian oedema advocate a strong degree of clinical suspicion when women in the reproductive age group present with painful abdomen, radiological evidence of multiple peripheral ovarian follicles in a solid ovarian tumour-like mass and normal biochemical tumour markers. Definitive surgical treatment should be undertaken only after confirmed pathological diagnosis [8, 28]. The role of frozen section needs to be emphasised in preventing unnecessary catastrophic reproductive outcomes. However, the chance of recurrence is a matter of concern although the chance is remote as discussed by Elkins *et al *[29]. The role of wedge resection, when massive ovarian oedema is suspected, involves removal of 30% or more of the ovarian volume to exclude secondary causes of the condition, and the possibility of adhesions needs to be weighed as against the complete removal of the ovary as a cause of fertility issues as discussed by Daboubi *et al *[28]. The successful treatment of massive ovarian oedema by laparoscopy is reported by various authors [30–33]. Laparoscopy with its double advantage of both diagnostic and therapeutic value is a possible option. If the ovary is enlarged and appears grey due to twisted pedicle and intact capsule, it would be prudent to detort the pedicle allowing the ovary to regain its vitality and then perform an ovarian biopsy followed by fixation of the ovary the posterior aspect of the uterus, which has been recommended in the presence of torsion, hence conserving the ovarian tissue and preventing a further episode of torsion. Following the conservative procedure, Hubbell *et al *[34] recommend the use of oral contraceptive therapy for a few months during the follow-up period, which may be beneficial in cases of massive ovarian oedema without the evidence of torsion.

Medical management would not be a practical option as the diagnosis of massive ovarian oedema is usually retrospective and there are no known medical methods available to manage this condition. A minimum of wedge resection with frozen section for confirmation of the diagnosis would be mandatory. Although wedge resection might negatively affect fertility at least in some cases due to the development of adhesions, this would be better than complete extirpation of the ovary. Hence, we were more conservative in our approach for the fourth woman in our study .We performed a frozen section and once the diagnosis of massive ovarian oedema was made, an ovarian decompression by ovarian drilling followed by wedge resection and reconstruction of the distorted ovary with redundant capsule close to normal was performed with great success.

## Conclusion

Massive ovarian oedema is a rare gynecologic condition in women in the reproductive age group. Due to the absence of pathognomonic clinical, radiological or biochemical characters, the role of intraoperative frozen section needs to be emphasised, which can guide a clinician to perform fertility-sparing procedures such as ovarian detorsion, ovarian drilling and decompression of the fluid, wedge resection, ovarian reconstruction with or without ovarian fixation procedures, hence preserve hormonal functions and fertility in these young women.

## Conflict of interest

The authors declare that they have no conflict of interest.

## Authors’ contribution

Dr. Praveen was involved in manuscript preparation, article design, analysis, patient care, reference hunting and final editing. Dr. Pallavi was involved with patient care and editing the manuscript. Dr Rajashekar was involved with patient care and editing the manuscript. Dr Usha was involved in histopathological diagnosis and reporting. Dr Uma Devi was involved with patient care and editing the manuscript. Dr Bafna played a key role in manuscript preparation, article design, the final corrections and editing the manuscript.

## Figures and Tables

**Figure 7:**
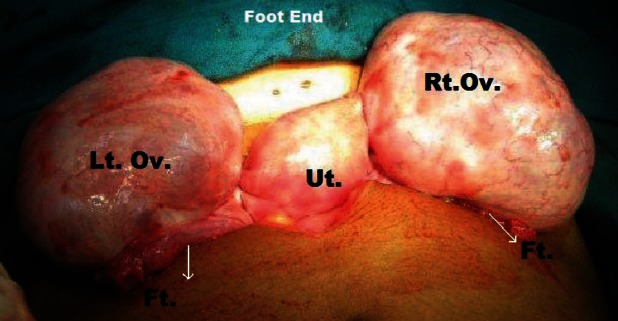
Intra-operative picture showing bilateral pearly white ovarian enlargement with multiple cysts and smooth shiny intact capsule.

**Figure 8:**
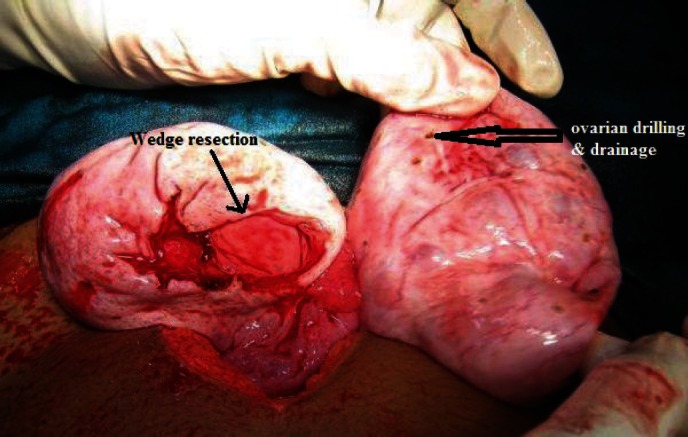
Wedge resection with bilateral ovarian drilling and drainage of the multiple cysts.

**Figure 9:**
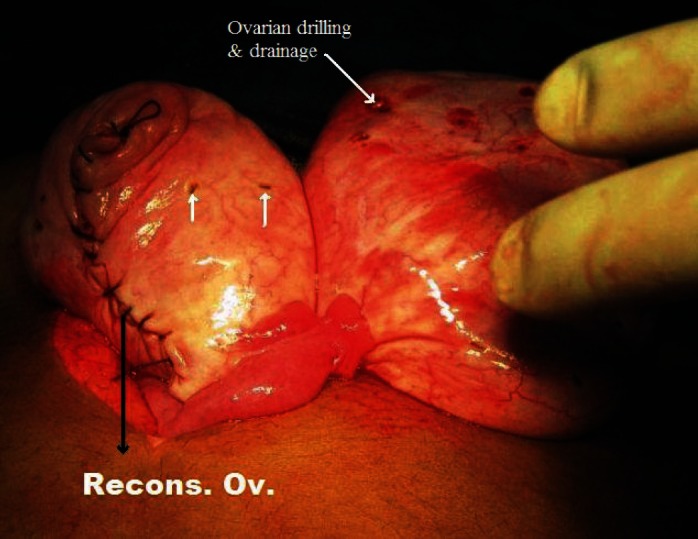
Ovarian reconstruction following wedge resection with ovarian drilling and drainage of the multiple cysts.

**Table 1:** Different modalities of treatment reported in the literature

Parameters	*n* = 177 (%)
**Types of intervention**	
SO^a^	145/177 (81.9%)
TAH+BSO^a^	12/177 (6.8%)
Fertility preserving surgery	20/177 (11.3%)
Wedge biopsy + FS ± ovarian suspension	14/20 (70%)
Diagnostic laparoscopy?	01/20 (05%)
Non-invasive radiologic monitoring (UGS/MRI) and symptomatic treatment	05/20 (25%)

SO, salpingo-oophorectomy; TAH + BSO, total abdominal hysterectomy and bilateral salpingo-oophorectomy; FS, frozensection; USG, ultrasound; MRI, magnetic resonance imaging.

a 88.7% of women did not have fertility-sparing surgery.

**Table 2:** Clinical characteristics.

Case no.	Age	Presenting symptoms		Clinical features		USG/MRI	*S-βhcg*, AFP, CA-125 and LDH
		Pre-menarcheal	Menarcheal	Symptoms	Mass /virilisation		
1	8	Yes	–	Pain abdomen	Bimanual palpable pelvic mass	Right ovarian multicystic mass, 8.2 × 3.2 × 5.2 cm (Figure 5)	Normal limits
2	11	–	Yes	Pain abdomen, Oligomenorrhoea	Hirsutism, obese, bimanual palpable abdomino-pelvic mass	Bilateral cystic and solid masses, 10 × 12 × 5 and 8 × 6 × 7cm of sizes	Normal limits
3	26		Yes	Pain abdomen	Bimanual palpable pelvic mass	Left adnexal cystic solid mass, 8 × 5 × 7 cm	Normal limits
4	17	–	Yes	Pain abdomen	Bimanual palpable pelvic mass	Bilateral cystic and solid masses, 8 × 6 × 6 and 6 × 6 × 7 cm of sizes	Normal limits

**Table 3:** The surgical details, histopathology and follow-up.

Case no.	Intra-op. findings	Procedure	Frozen and Final HPR	Final treatment decision
1	A right ovarian soft fleshy oblong mass of 8 × 5. × 4 cm and intact capsule with torsion and no normal ovarian tissue was seen	Right salpingo-oophorectomy with omental biopsy and peritoneal cytology	Massive ovarian oedema	On clinical follow-up
2	Right adnexa revealed two cystic-solid masses of size 15 × 15 and 8 × 6 cm, intact capsule, twisted twice and no normal ovarian tissue was seen. The left adnexa revealed 5 × 6 cm cystic-solid mass with intact capsule	Right oophorectomy, left ovarian cystectomy with ovarian reconstruction with omentectomy and peritoneal cytology	Massive ovarian oedema	On hormone replacement therapy
3	A left ovarian solid-cystic, intact capsule mass of size approximately 8 × 5 × 4 cm, without a torsion	Left oophorectomy with omental biopsy and peritoneal cytology	Massive ovarian oedema	On clinical follow-up
4	Bilateral ovaries showed pearly white enlargement with multiple cysts and smooth shiny intact capsule. The left ovary was 8 × 6 × 6 cm and right ovary 6 × 6 × 6 cm in size, and there was no torsion noticed (Figure 7)	Wedge resection with bilateral ovarian drilling with drainage of the multiple cysts and ovarian reconstruction (Figures 8 and 9)	Massive ovarian oedema and a corpus luteal cyst	On clinical follow-up and oral contraceptive pills given for 6 months
